# Feature fusion based VGGFusionNet model to detect COVID-19 patients utilizing computed tomography scan images

**DOI:** 10.1038/s41598-022-25539-x

**Published:** 2022-12-16

**Authors:** Khandaker Mohammad Mohi Uddin, Samrat Kumar Dey, Hafiz Md. Hasan Babu, Rafid Mostafiz, Shahadat Uddin, Watshara Shoombuatong, Mohammad Ali Moni

**Affiliations:** 1 Department of Computer Science and Engineering, Dhaka International University, Dhaka, 1205 Bangladesh; 2 School of Science and Technology, Bangladesh Open University, Gazipur, 1705 Bangladesh; 3 Department of Computer Science and Engineering, University of Dhaka, Dhaka, 1000 Bangladesh; 4 Institute of Information Technology, Noakhali Science and Technology University, Noakhali, 3814 Bangladesh; 5 School of Project Management, Faculty of Engineering, The University of Sydney, Forest Lodge, NSW 2037 Australia; 6 Center of Data Mining and Biomedical Informatics, Faculty of Medical Technology, Mahidol University, Bangkok, 10700 Thailand; 7 Artificial Intelligence and Digital Health Data Science, School of Health and Rehabilitation Sciences, Faculty of Health and Behavioural Sciences, The University of Queensland, St Lucia, QLD 4072 Australia

**Keywords:** Medical imaging, Computational science, Computer science, Information technology

## Abstract

COVID-19 is one of the most life-threatening and dangerous diseases caused by the novel Coronavirus, which has already afflicted a larger human community worldwide. This pandemic disease recovery is possible if detected in the early stage. We proposed an automated deep learning approach from Computed Tomography (CT) scan images to detect COVID-19 positive patients by following a four-phase paradigm for COVID-19 detection: preprocess the CT scan images; remove noise from test image by using anisotropic diffusion techniques; make a different segment for the preprocessed images; and train and test COVID-19 detection using Convolutional Neural Network (CNN) models. This study employed well-known pre-trained models, including AlexNet, ResNet50, VGG16 and VGG19 to evaluate experiments. 80% of images are used to train the network in the detection process, while the remaining 20% are used to test it. The result of the experiment evaluation confirmed that the VGG19 pre-trained CNN model achieved better accuracy (98.06%). We used 4861 real-life COVID-19 CT images for experiment purposes, including 3068 positive and 1793 negative images. These images were acquired from a hospital in Sao Paulo, Brazil and two other different data sources. Our proposed method revealed very high accuracy and, therefore, can be used as an assistant to help professionals detect COVID-19 patients accurately.

## Introduction

COVID-19^1^, also known as the Coronavirus, was spread in Wuhan, China. Within four months of its emergence in the year 2020, the World Health Organization (WHO) declared it a pandemic^2–4^. Confirmed cases and deaths are recorded as 538,246,806 and 6,327,036, respectively, and these numbers are increasing day by day and until June 2022, 230 countries are currently being affected by COVID-19^5^.

As early COVID-19 symptoms, sometimes patients feel difficulty in breathing and do vomiting. Sneeze and cough droplets from an infected individual can easily spread from one person to another. With the large number of patients infected by COVID-19 during the pandemic, it was impossible for health experts and the competent authorities to assure enough testing kits for each. Besides, there is a shortage of kits to find out infected people^6^. If more tests occur, it gets easy to find out more COVID-19 affected people and help not spread Coronavirus. One of the most used methods for diagnosing COVID-19 instances is reverse transcription polymerase chain reaction (RT-PCR), where respiratory samples are used to perform the test. RT-PCR can provide the result to the patients at a minimum time. However, it does not take the minimum time in most cases, and it almost takes two days to three days. Blood samples are also used to detect Coronavirus; here, Antibodies from the blood are helped to detect COVID-19.

In recent times, two different methods have gained popularity in detecting COVID-19 one is from X-ray images, and another is from the computerized tomography (CT) images. In Wuhan, there was a study performed by different authors^7^, from the study, we have obtained that utilizing CT images COVID-19 infection rate was about 98% and sensitivity was 71.5%. When infected people arose, Chinese clinical centers had insufficient testing kits. Therefore, medical professionals started using chest CT images to identify the COVID infected people. Due to the inadequate number of testing kits, Turkey already used CT images to detect COVID-19 infected people^8,9^. Studies performed by the authors^10,11^ showed that early identification of COVID-19 is improved by clinical imaging characteristics.

Furthermore, it is noticed by health professionals that the changes can be detected in chest CT images before the symptoms are visible^12^. Compared to X-ray images, CT images contain significantly better image quality, image details and a three-dimensional view of the lung. This is another prime reason for choosing CT images to detect COVID-19 positive and negative in this exploration. At present, machine learning techniques are widely used to detect specific features and conditions in imaging. Deep Neural Network is one of the best techniques of machine learning used for the classification of skin cancer^13^, breast cancer detection^14^, X-ray of the chest for pneumonia detection^15^, and lung segmentation^16^. Thus, to detect COVID-19 infected people, faster and more intelligent detection models can help the medical professional. In this paper, a Convolutional Neural Network (CNN) based deep approach is proposed to detect COVID-19 infected people from the CT images. This research has utilized three open research datasets available at different repositories. After combining the three data sets, we have 3068 positive and 1793 negative COVID-19 CT images. Another important expectation of our research is to use the feature extraction technique. A combined CNN and Histogram of Oriented Gradients (HOG) principle is used to propose our model in this framework. In a nutshell, our contributions to this paper are threefold:

In our proposed system, anisotropic diffusion is utilized to remove the noise from the CT images. Segmentation is used to detect the significant fracture regions of lung cells. Then the HOG and the CNN features extraction are performed separately. Finally, both features are combined into a feature vector map. Feature vector maps have been utilized to achieve further classifications. Furthermore, the CNN classifier classifies COVID-19 into positive or negative classes.

## Relevant works

Detection and classification of COVID-19 using a deep task neural network is challenging. Many researchers have used several methodologies or techniques to detect COVID-19. In this section, some recent approaches used to detect COVID-19 are described. At present, many research works have been conducted using deep learning models to detect pneumonia, cancer, brain tumour, skin diseases from MRI images, X-ray images or CT images. Varshni et al.^17^ proposed a CNN-based model to detect pneumonia. They achieved better accuracy using DesNet-169 as a feature extraction technique. Ling et al.^18^ utilized CT images to detect pneumonia using the LDA-SVM Classification technique. For filtering, the authors used median binary and closed operation. For feature extraction, they used Added Local Binary Pattern (LBP) feature, HOG and texture. They achieved an accuracy of 92.77% for LDA-SVM, 91% for only SVM, 85% for Adaboost, and 83% for Linear Discriminant Analysis (LDA). Wu et al.^19^ proposed a model to detect COVID-19 based on multi-view fusion. In that proposed framework, they used ResNet50 as a variant of CNN. The authors used 495 CT images where 368 were COVID positive, and 127 were other Pneumonia. These CT images are collected from the hospitals in China. In that framework, 80%, 10%, and 10% of the dataset were used for training, testing, and validation. This system achieves 76% accuracy with 81.9% Area under Curve (AUC). Yousefzadeh et al*.*^20^ developed a deep learning model named ai-corona to detect COVID-19 utilizing CT images. Different models of CNN titled ResNet, DenseNet, EffcientNetB0, and Xception are used to form this framework. The authors used a dataset of 2124 CT slices where the number of non-COVID-19 images was 1418 and COVID-19 infected images was 706. The dataset was plunged into a portion of 20% and 80% for validation and training, respectively. 96.4% accuracy was found in this proposed framework with 92.4% sensitivity and 98.3% specificity.

An artificial intelligence-based diagnosis system has been proposed by Jin et al.^21^ to detect COVID-19. In this developed system, ResNet152 is used as a variant of CNN, and the dataset was obtained from two freely accessible databases and three eminent Chinese clinics. The dataset contains 1881 CT images where 1385 images of COVID-19 negative cases and 496 COVID-19 positive cases. From the experiment of the proposed system, the authors obtained 94.98% accuracy with 94.06% sensitivity and 91.53% precision. Furthermore, Xu et al.^21^ introduced a system to classify Influenza-A viral pneumonia and COVID-19 using CNN. Resnet18 pre-trained model was used in that system as a variant of CNN. Total 618 CT images were used in their work, where the number of COVID-19 infected images was 219, Influenza-An infected images were 224, and 175 were normal CT images. In another research, Ardakani et al*.*^22^ used ten different variants of CNN to detect COVID-19 utilizing CT images. The proposed model's dataset consisted of 1020 CT images. Xception and ResNet-101 architecture showed better performance than others from the ten variants. Using the ResNet-101 model, the authors obtained 99.51% accuracy and 100% sensitivity. While using Xception, the authors found 99.02% accuracy and 98.04% sensitivity. Liu et al*.*^23^ proposed an automatic diagnosis system to detect COVID-19 utilizing deep learning and CT images. Modified DenseNet-264 was used in that system for the diagnosis where four dense blocks were used. For this experiment, the dataset consisted of 920 COVID-19 positives and 1073 non-COVID-19 CT images. 60% of images from the dataset were used in the training phase, and for the testing, and validation 20% of images were used individually. Ying et al*.*^24^ proposed a deep learning technique named Deep Pneumonia to detect COVID-19 utilizing CT images. This developed system is based on a Relation Extraction neural network (DRE-Net), where Feature Pyramid Network and ResNet50 are used. The dataset was formed with 1990 CT image slices were 777, 505, and 708 CT image slices of COVID-19 positive, bacterial Pneumonia, and normal, respectively. For the training phase, the authors used 60% CT images of the dataset, and for testing and validation, 30% and 10% images were used individually. The authors obtained 94% accuracy, 96% precision and 93% sensitivity.

A hybrid system has been proposed by Hasan et al*.*^25^ for COVID-19 diagnosis in healthy people and pneumonia cases utilizing CT images and the concept of deep learning features and Q-deformed entropy. In this proposed system for feature extraction, Q-deformed entropy and CNN were used, and for the classification, LSTM was used. The dataset consists of 321 chest CT images containing 118 images of COVID-19 cases, 96 images of pneumonia cases, and 107 healthy individuals. The authors achieved 99.68% accuracy. Another deep learning method has been developed by Amyar et al*.*^26^, utilizing CT samples to diagnose COVID-19 patients. In this system, an encoder was used for the reconstruction, two decoders were used for segmentation, and a multi-layer perceptron was utilized for the classification. The system achieved 86% accuracy, 94% sensitivity, and 79% specificity.

## Proposed methodology

**Figure 1:**
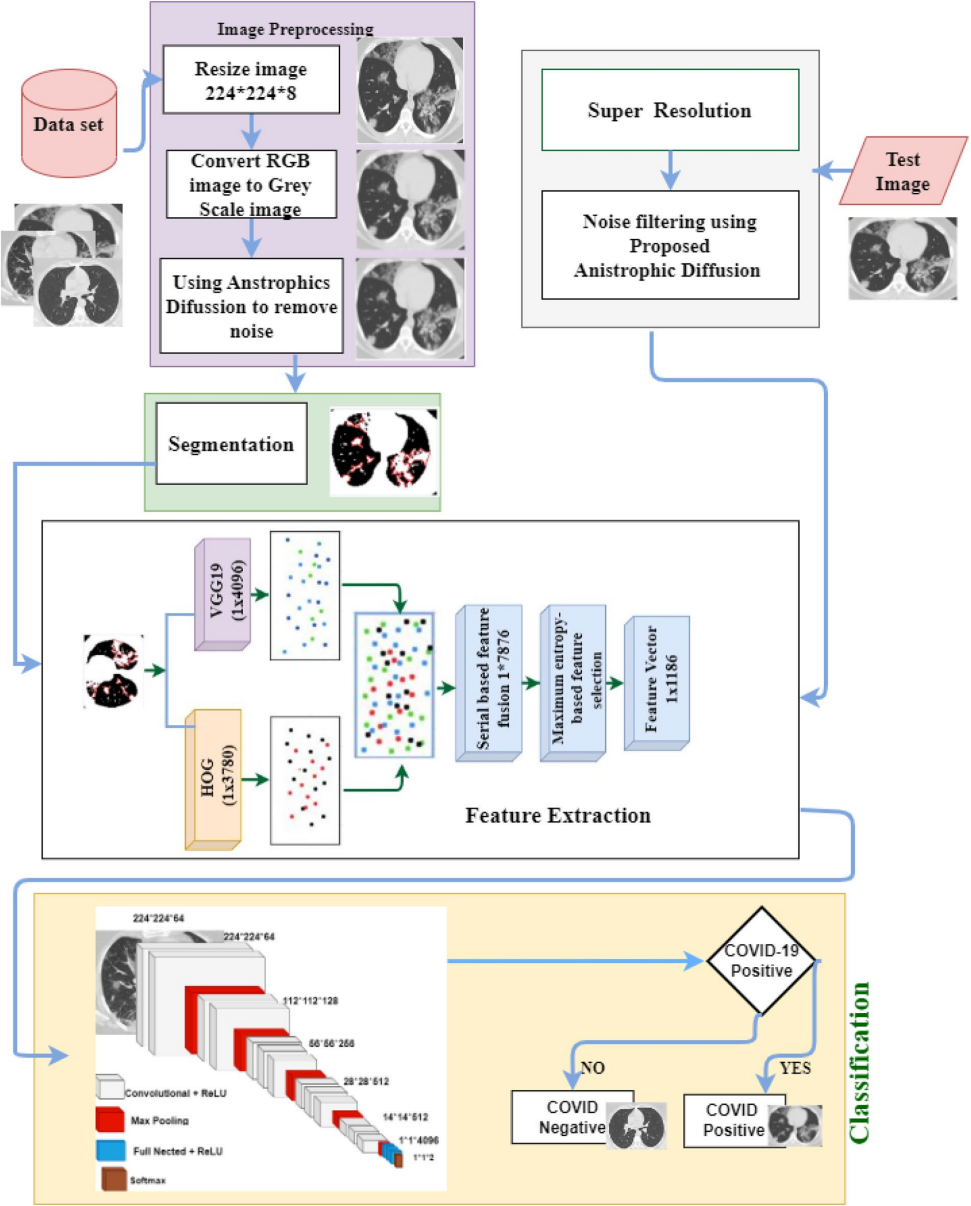
Workflow diagram of proposed VGGFusionNet model.


The proposed methods are applied to the three publicly available datasets that are dedicatedly focused on the collections of CT scan images of COVID and non-COVID patients. Informed consent was received from each patient during the development of those datasets by the respective authors of each dataset. This study only utilized those accessible datasets and proposed a novel method to detect COVID-19 from CT scan images. The literature^27–29^ states that all techniques utilized to gather data and create a dataset centered on CT scan images were carried out in conformity with pertinent rules and regulations. In addition, the proposed methodology (VGGFusionNet) was conducted in accordance with the Declaration of Helsinki. This section shows the Computer Vision (CV) approach to detect COVID-19 positive and negative people from CT images. Several CT images, including COVID-19 positive and negative, were utilized to train the VGGFusionNet proposed model. The sample data for the training phase contains a high level of quality and contrast. For detecting Region of Interest (ROI), unwanted regions are removed, and before doing this, the grayscale conversion has been processed for all the input datasets. The patches are then accumulated from the ROI images, and then filtering and segmentation methods are performed. For feature extraction, CNN and HOG are combined to create feature vectors. Finally, the classification is performed using these feature vectors. The test image is then categorized according to whether or not it is influenced by COVID-19. Figure 1 depicts the proposed system's functioning method and is also represented by Algorithm 1. This model has been used for each training image to apply preprocessing techniques and feature extraction using CNN with *O*(*n*) complexity. HOG feature extraction technique has been performed for each image with *O*(*n*) complexity. This model produces the complexity [*O*(*n*) + *O*(*n*)] for the *n* number of an input image.

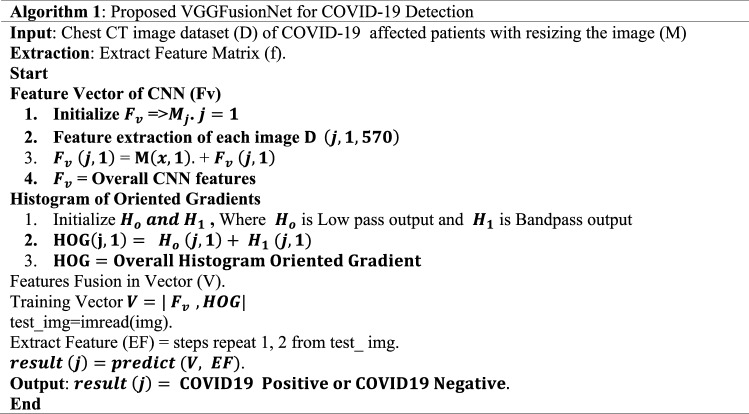


### Dataset

The patients' CT scan images have been collected and stored in a data repository. To evaluate the proposed intelligent system's performance, the images were divided into two categories: normal and COVID-19. The performance of the system was validated using three standard datasets in this study.

(A) The data set^27^ utilized in this experimental assessment comprised of 1600 COVID-19 and 100 normal CT scan images in two primary categories.
(B) The COVID-CT-Dataset proposed by Yang et al*.*^28^ from the Cornel University have used CT images from 216 patients, with 349 COVID-19 CT images and 463 non-COVID-19 CT images, respectively.
(C) In this article^29^, a publicly accessible CT scan dataset of 2482 CT scans, containing 1252 CT COVID-19 positive and 1230 COVID-19 negative.

**Figure 2:**
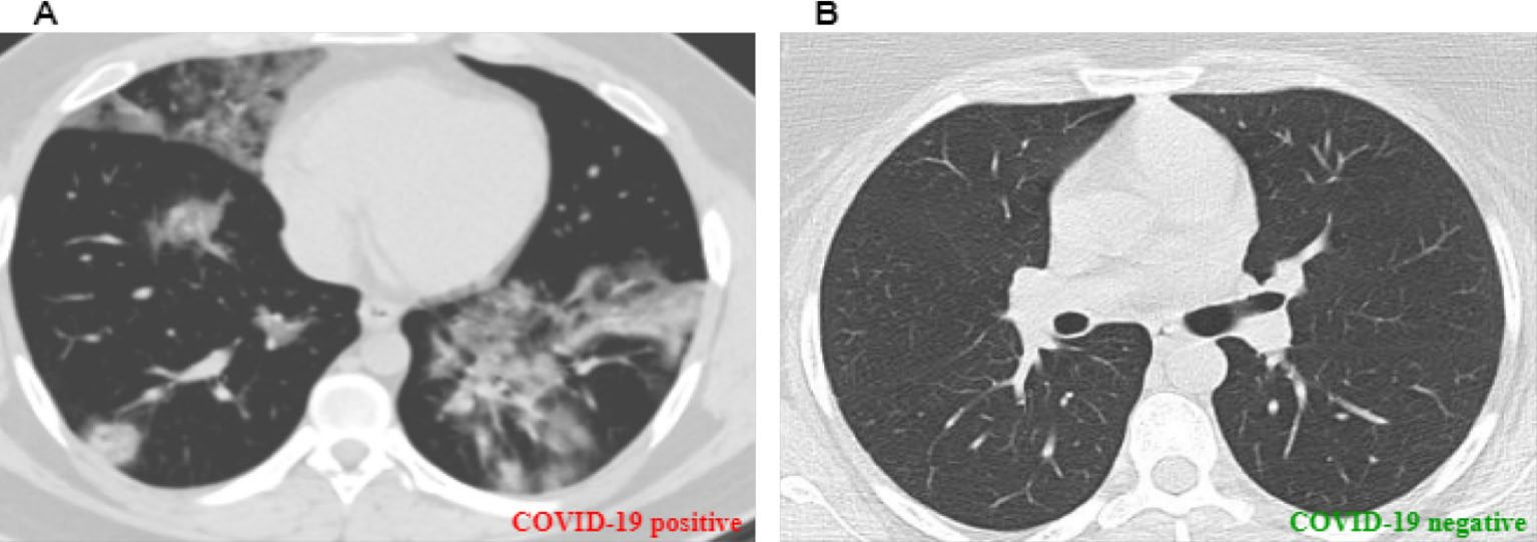
CT scan images of COVID-19 positive and negative.

The images from all three data sets were merged to generate a dataset for this work because the number of images accessible in the public repository was limited. Both COVID-19 and normal classes were used in the training and testing stages. The COVID-19 class contained 3068 images, and the class of the normal image included 1793 images. The entire dataset was divided into 0.8 portions for training and the remaining 0.2 portions for testing based on the patients’ distribution. This experiment considered individual patients as distinct train data rather than using random CT images. This will ensure unbiased results as two very similar samples will not be present in the training phase along with the testing phase also. Figure 2 shows a comparison between COVID-19 and normal CT scan images. In the case of COVID-19, the lung density is increased, which causes whiteness in the lungs on radiography, similar to cases of Pneumonia.

### Data pre-processing

**Figure 3:**
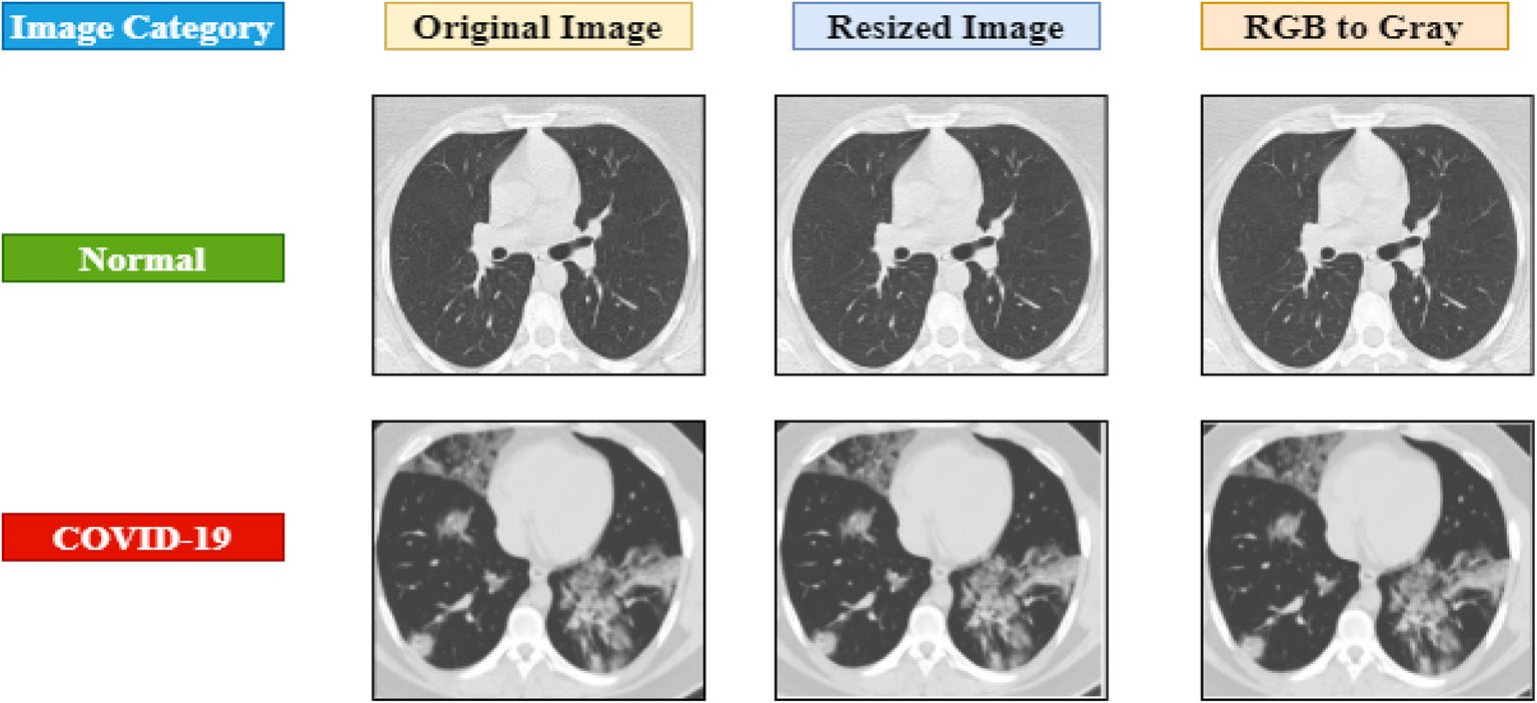
Image preprocessing stages.

Image processing is a crucial step in obtaining meaningful information and correct classification. The images were converted from RGB to grayscale utilizing a MATLAB tool and resized to 224 × 224 pixels before being fed into the algorithm. Figure 3 shows examples of images at various stages of data preprocessing.

### Modified anisotropic diffusion filtering (MADF)

Filtering techniques keep the relevant information in an image while removing any noise. Information-preserving filtering algorithms are the most effective at extracting significant features from any noisy image. Filtering performance has been evaluated using speckle-affected test images during the testing phase. Edge information can be preserved and enhanced while noise is suppressed using anisotropic diffusion filtering. The edge information, as well as noise, is detected by the gradient operator. For severe speckle and low contrast images, this method detects noise gradient changes beyond the edge gradient. These alterations remove edge information more than noise, resulting in worse filtering accuracy.

Similarly, owing to image smoothing, speckle reducing anisotropic diffusion cannot retain all of the edge information. Oriented-based non-local methods (OBNLM) are affected by moving noise and are unable to store precise information. Anisotropic diffusion with memory-based speckle statistic (ADMSS) is crisper^30^. For this study, MADF has been recommended as a way to maintain precise information while reducing image noise and distortion. This filtering technique outperforms the others because of its capacity to eliminate multiplicative speckle noise in-plane regions. To store important edge information, the proposed method employs correlation and noise kurtosis values. This speckle suppression procedure is repeated until the image's noise component approaches Gaussian values. The kurtosis should be zero in this circumstance. Equation (1) represents the noise part, and iteration continues until the noise part's kurtosis falls below the values computed using Eq. (3). Equation (2) can be used to define this measurement. The loop ends when the slightest correlation between image class and noise class. From Eqs. (1) to (7)^31^, I and I_0_ denote the actual and noisy image, respectively, and G indicates the noise intensity means. The kurtosis value k is found using Eq. (5). Equation (6) calculates the image intensity correlation, while Eq. (7) calculates the noise intensity correlation. When ρI and ρG have the lowest deviation, the proposed filtering produces the best results.

1 $${I}_{0}={I}_{n}$$

2 $$\mathrm{n}= \frac{\mathrm{I}-\mathrm{G}}{\surd \mathrm{G}}$$

3 $$\upmu =\frac{{\sum }_{i=1}^{N}{G}_{i}}{N}$$

4 $$k=\frac{\frac{1}{N} {\sum }_{i=0}^{N}{\left(G-\upmu \right)}^{4}}{{[\frac{1}{N} {\sum }_{i=0}^{N}{\left(G-\upmu \right)}^{2}]}^{2}}-3$$

5 $$\mathrm{abs}(\mathrm{n}-\mathrm{k})\le 0.001$$

6 $${\uprho }_{\mathrm{I }}=\frac{{\sum }_{\mathrm{i}=0}^{\mathrm{M}-1}{\sum }_{\mathrm{j}=0}^{\mathrm{N}-1}\mathrm{i}.\mathrm{j}.{\mathrm{p}}_{\mathrm{I }}\left(\mathrm{i},\mathrm{j}\right)-\upmu {\mathrm{I}}_{\mathrm{x}}\upmu {\mathrm{I}}_{\mathrm{y}}}{\frac{\sum_{\mathrm{i}=1}^{\mathrm{N}}({\mathrm{I}}_{\mathrm{ix}}-{\upmu }_{\mathrm{Ix}}){(\mathrm{I}}_{\mathrm{iy}}-{\upmu }_{\mathrm{Iy}})}{\mathrm{N}}}$$

7 $${\uprho }_{\mathrm{G }}=\frac{{\sum }_{\mathrm{i}=0}^{\mathrm{M}-1}{\sum }_{\mathrm{j}=0}^{\mathrm{N}-1}\mathrm{i}.\mathrm{j}.{\mathrm{p}}_{\mathrm{G }}\left(\mathrm{i},\mathrm{j}\right)-\upmu {\mathrm{G}}_{\mathrm{x}}\upmu {\mathrm{G}}_{\mathrm{y}}}{\frac{\sum_{\mathrm{i}=1}^{\mathrm{N}}({\mathrm{G}}_{\mathrm{ix}}-{\upmu }_{\mathrm{Ix}}){(\mathrm{G}}_{\mathrm{iy}}-{\upmu }_{\mathrm{Iy}})}{\mathrm{N}}}$$

**Figure 4:**
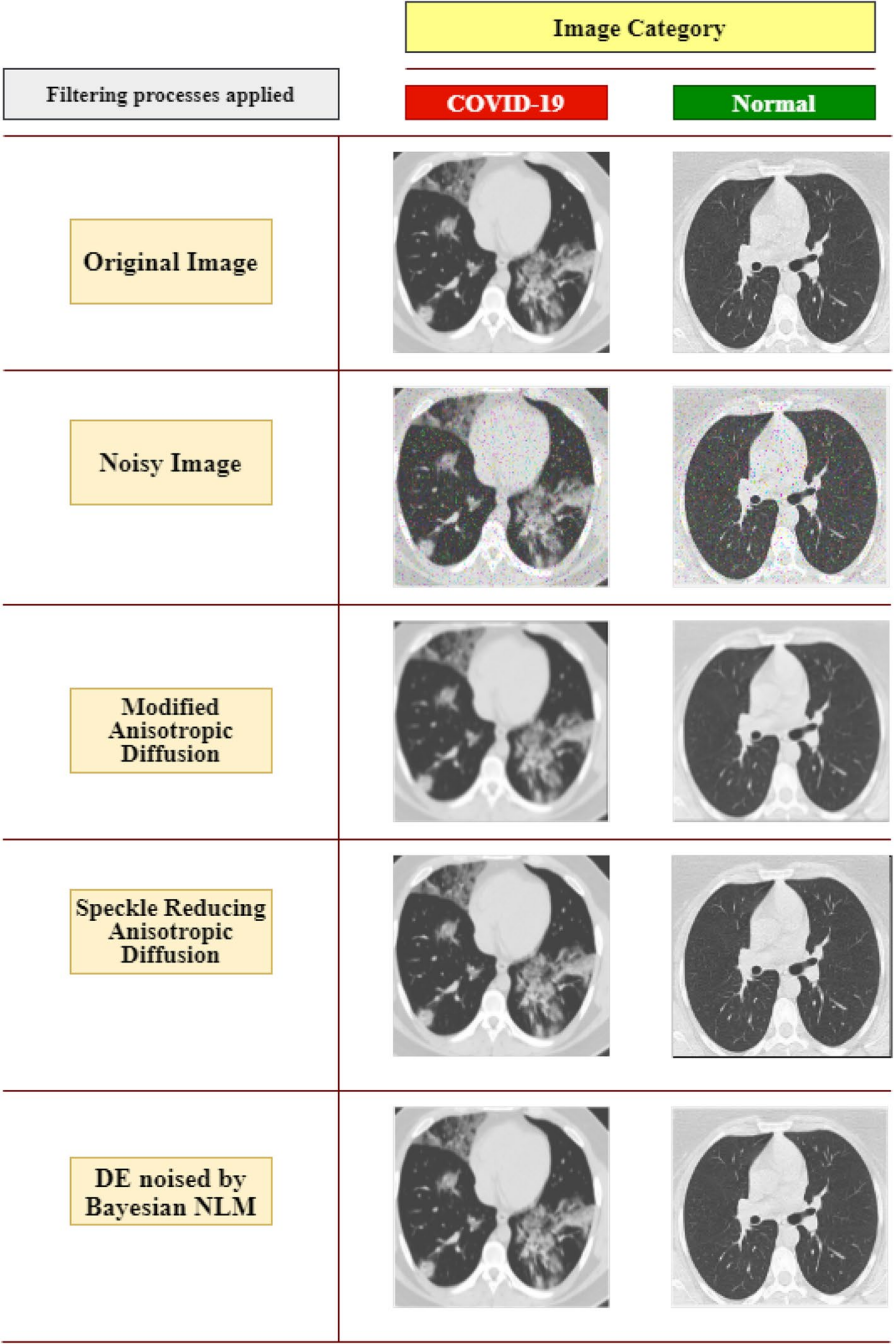
Category wise (COVID-19 and normal) images after different diffusion techniques.

A comparison of several Anisotropic Diffusion methods is shown in Fig. 4 and an example of original images. The proposed MADF technique's edge preservation capability was superior to the other techniques.

### Image segmentation

Segmentation is the non-trivial task for differentiating the individual pixels of computed tomography (CT) images. The segmented images would only be considered for the following processes. A designed segmentation technique simplifies the chest CT data for its less relative computational complexity. The applied segmentation method provides an improved detection accuracy for the infected image. After investigating preprocessed data, the experiment found that the desired result was obtained for two groups of clusters. The fracture regions from the COVID-19 CT scan images. Each iteration updates the cluster's centroid to reduce the distance between each intensity and the centroid^32^. The centroid is calculated by the mean intensities of all pixels within a group of clusters using Eq. (8). The distance is calculated using Eq. (9).

8 $${C}_{k}=\sum {z}_{i}/ N $$

9 $$r=|{C}_{k}-{x}_{i}|$$

**Figure 5:**
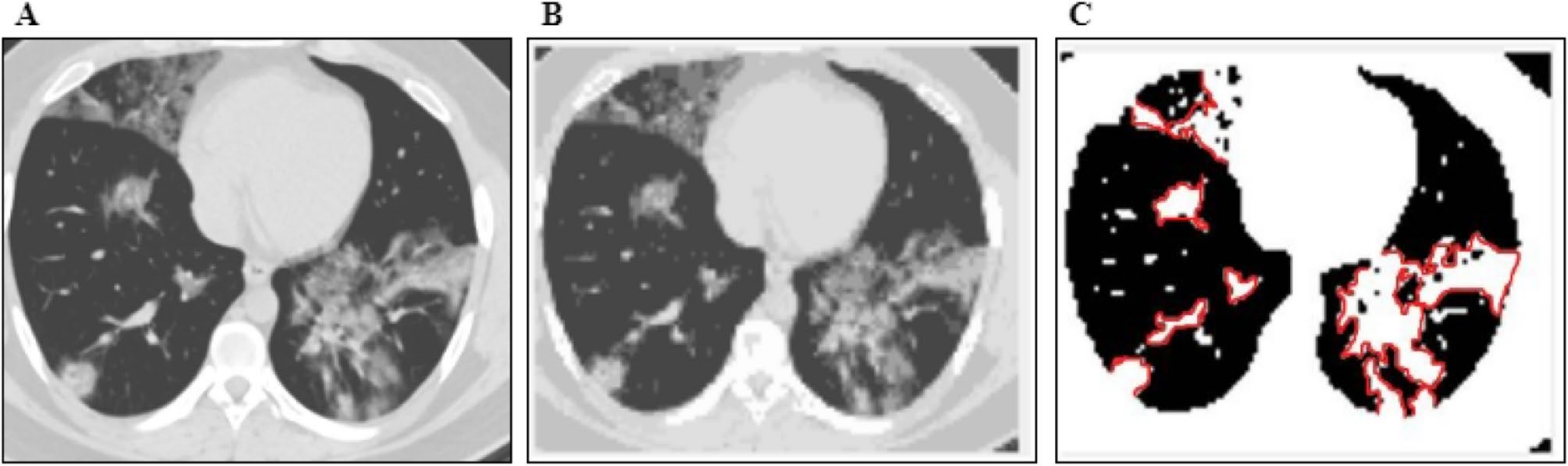
Image segmentation stages using region segmentation algorithm.

Here the centroid of $k$th cluster is defined as ${C}_{k}$ and the within-cluster intensity is ${z}_{i}$. ${x}_{i}$ is denoted for each pixel intensity for the grey level of $N$. This experiment has used the clustering technique for region segmentation. Figure 5 shows the results of segmenting a sample image using Algorithm 2^33^.
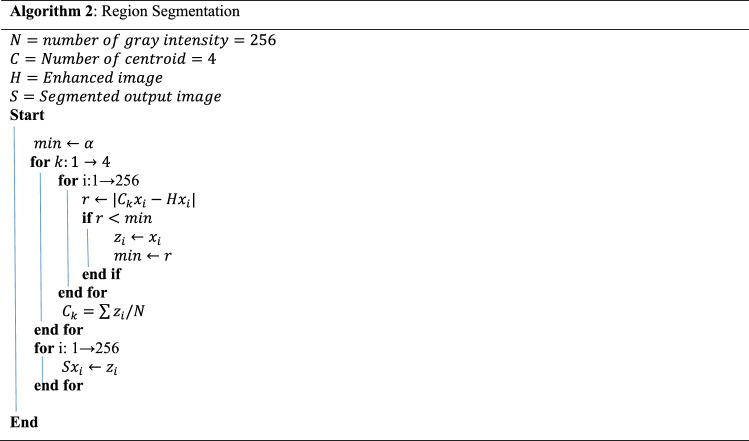

### Feature extractor

Two feature extraction approaches, Histogram Oriented Gradient (HOG) and VGG-19 are used individually in this study. These features are incorporated into a feature vector when obtained using HOG and VGG-19. This fused vector aids in detecting COVID-19 positivity or negativity from a CT scan with the most remarkable accuracy.

#### Histogram oriented gradient (HOG)

The exploratory analysis found that our experiment's HOG^34^ feature descriptor is more robust for chest CT data. With the use of local intensity gradients, the HOG feature extractor can characterize object information^34^. The image is decomposed into smaller gradients, and these parts are combined. A Sobel kernel is used to filter the segmented image to obtain the gradient direction of ${G}_{x}$ and ${G}_{y}$. Equations (10) and (11) calculate the magnitude of angle and gradient at each pixel.

10 $${f}_{|G\left(i,j\right)|}=\sqrt{{G}_{x}{(i,j)}^{2}+{G}_{y}{(i,j)}^{2}}$$

11 $${\theta }_{|G\left(i,j\right)|}={\mathrm{tan}}^{-1}(\frac{{G}_{y}(i,j)}{{G}_{x}(i,j)})$$

Here the $f$ is the gradient magnitude at direction $\theta$ for the pixel denoted by row $i$ and column $j$. Then, a histogram is found using the angle and gradient. The normalization vector is formed using each block of the histogram. Finally, Eq. (12) represents the HOG feature descriptor with eight block sizes,

12 $$V=\sum_{i}^{N}\frac{{V}_{i}}{\sqrt{{V}_{i}^{2}+k}}$$

The normalized vector is combined in each block, and the HOG feature vector is obtained using Eq. (12). It differentiates the significant region of an image patch containing useful information by forming a histogram and skipping the extraneous values. HOG is a robust feature generator for object recognition in the image. It is much easier and quicker to compute. Furthermore, HOG describes a whole image or patch, whereas SIFT^35^ or other features extraction techniques describe a specific point in the image. It also provides more reliable features than other feature extraction techniques.

**Figure 6:**
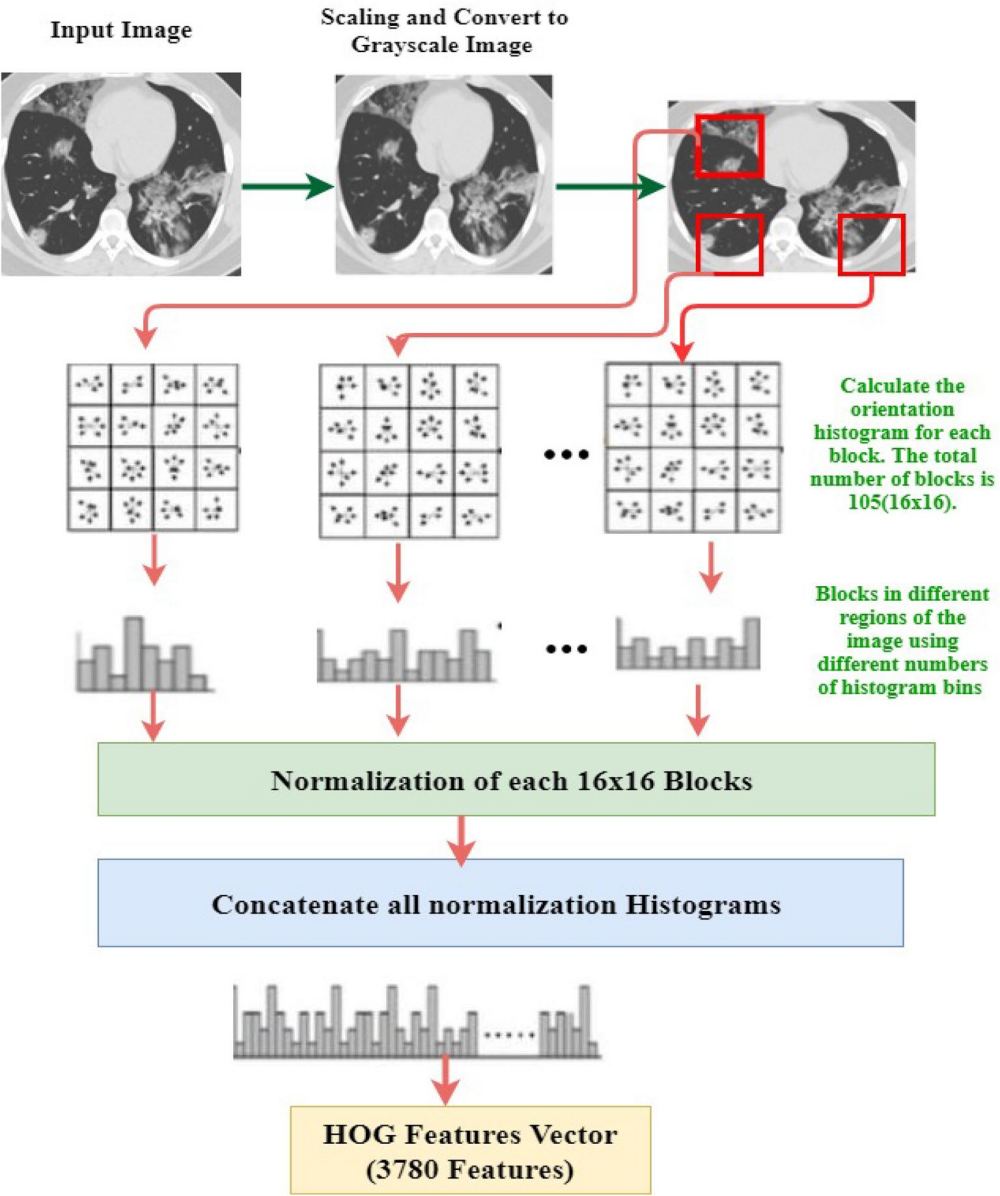
Architectural representation of histogram oriented gradient features extraction approach.

For HOG feature extraction, the images are preprocessed into size 64 × 128. This can be achieved by the image patches of 8 × 8 and 16 × 16, respectively. Then, the gradient is calculated for each image pixel in $x$ and $y$-direction. A calculated gradient helps to obtain the pixel values for each patch. The exact process is repeated for all the pixels in the image. By doing so, continue to the whole image; the features are recorded for each smaller patch of the image. While dividing the image into 88% cells, this experiment generates a 9 × 1 matrix for each cell. Finally, a 16 × 16 block forms by combining four 8 × 8 cells. As the histogram of each 8 × 8 cell performs one 9 × 1 matrix, the total 16 × 16 block has one single 36 × 1 vector. In Eq. (5), the normalized vector size would be 36 × 1. The gradient of this normalized vector in the horizontal position is 7 and in the vertical position is 15 forming a total of 7 × 15 = 105. Thus, this experiment has found total features for each image would be 105 × 36 × 1 = 3780 using the HOG technique. The overall HOG features extraction approach is depicted in Fig. 6.

#### CNN based feature extractor and classification

One of the most prominent advancements in computer vision is image feature extraction utilizing CNN. Several CNN models, both from scratch and pre-trained, were used for the test in this experiment. While the scratch model performs badly with a little datasets, the pre-trained model assists in reducing the quantity of data needed. As a feature extractor, we fine-tune a pre-trained VGG19 model using our experiment dataset. The 19-layer version of VGGNet was used to create this network model. In this experiment, VGG19 outperformed VGG16, as well as other deep learning models including Alexnet, ResNet50, and scratch models.

**Figure 7:**
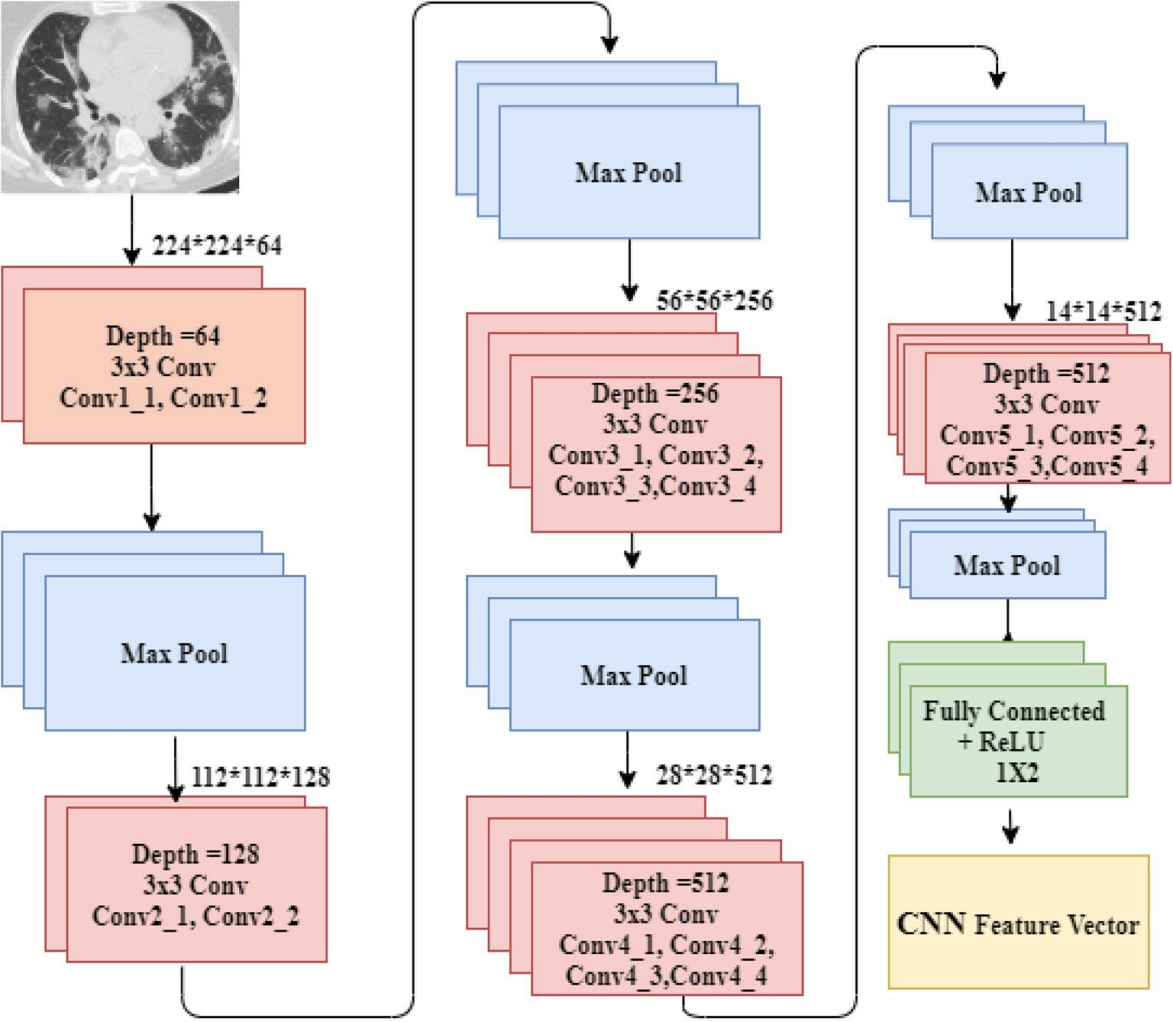
Architecture of the VGG19 model.

The VGG19 model has sixteen convolution layers followed by three fully connected layers, shown in Fig. 7. For each convolution layer's output, a non-linear ReLU is employed as an activation function. The whole convolution parts are divided into five sub-regions by five successive max-pooling layers. Two convolution layers, 64 and 128 respectively, make up the first and second sub-regions.

The remaining three sub-regions are made up of four convolution layers with values of 256, 512, and 512. After the convolutional layers' sub-regions, pooling layers are used to minimize the learnable parameter. Our VGG19 model's final layer is replaced with a softmax classification layer. Before the sigmoid output function, two hidden layers with 512 and 4096 neurons are included. To avoid overfitting in the execution of this fine-tuned model, L2 regularization was used after each fully connected layer except the dropout layer.

In the literature, data fusion has been employed in a number of computer vision and machine learning applications. 14,096 and 13,780 feature vectors are provided by two feature extractors. HOG and CNN features are retrieved from Eqs. (13) and (14), respectively. Equation (15) represents the fusion of the extracted features into a single vector.

13 $${f}_{HOG 1xn}=\{ {HOG}_{1x1}, {HOG}_{1x2}, {HOG}_{1x3}\dots \dots \dots \dots \dots \dots {HOG}_{1xn}\}$$

14 $${f}_{VGG-19 1xm}=\{ {VGG19}_{1x1}, {VGG19}_{1x2}, {VGG19}_{1x3}\dots \dots \dots \dots \dots \dots {VGG19}_{1xm}\}$$

15 $${Fused(features vector)}_{1xq} = \sum_{i=1}^{3}\{{f}_{HOG 1xn}, {f}_{VGG-19 1xm}\}$$

The presented method uses entropy-based feature fusion with a fused vector of 1 × 1186. Entropy has been applied to the feature vector to choose optimal features based on score values. Equations (16) and (17) reflect probability features and entropy, respectively. Equation (17) has been used to illustrate the feature selection process^37^ mathematically. From 7876 features, 1186 score-based features have been chosen using entropy. The classifiers were fed the last selected features to recognize COVID-19 images,

16 $${B}_{He}= -N{He}_{b}{\sum }_{i=1}^{n}p(f)$$

17 $${F}_{select}= {B}_{He}(\mathrm{max}({f}_{i, }1186))$$

In Eqs. (16) and (17), *p* stands for probability of features, and *He* stands for entropy. The classifiers are provided with the features that have been chosen at the end. The proposed method has been tested on a fused features vector, including HOG and deep learning features. The VGG-19 architecture consists of 16 CNN layers, three fully linked layers, and a softmax function layer. The fully connected and last layers remain the same for all network architectures. Max pooling was performed over 2 × 2-pixel windows with stride 2. The first two layers yield 4096 features out of the three completely linked layers, and the third layer offers 1000 channels. With two neurons, the last layer represents the result (COVID-19 and Normal).

## Results

In this research, accuracy, specificity, sensitivity, precision parameters are used for evaluating the computational result, and these parameters are achieved by using True Positive (TP), True Negative (TN), False Positive (FP) and False Negative (FN) which are derived from the confusion matrix. The computing formula from the value of the confusion matrix of these four performance parameters is Accuracy (ACC), Specificity (SPEC), Sensitivity (SEN), and Precision (PREC). The tests were carried out using a local machine platform that met the following requirements (Table 1). The proposed VGGFusionNet model was trained with different hyperparameters of the deep learning model. With a learning rate of 0.001 and batch size of 64, the Adam optimizer was utilized with 25 epochs. The Softmax activation function was employed in the experiment during the training of the model.

**Table 1:** Specifications of the system.

System specifications for the VGGFusionNet model development	
RAM	16 GB
CPU	1 × single core hyper threaded; Xeon processors @2.3 GHz
Cache	46 MB
GPU	NVidia K80 GPU
GPU memory	16 GB
Session limit	10 h
Disk space	100 GB

**Figure 8:**
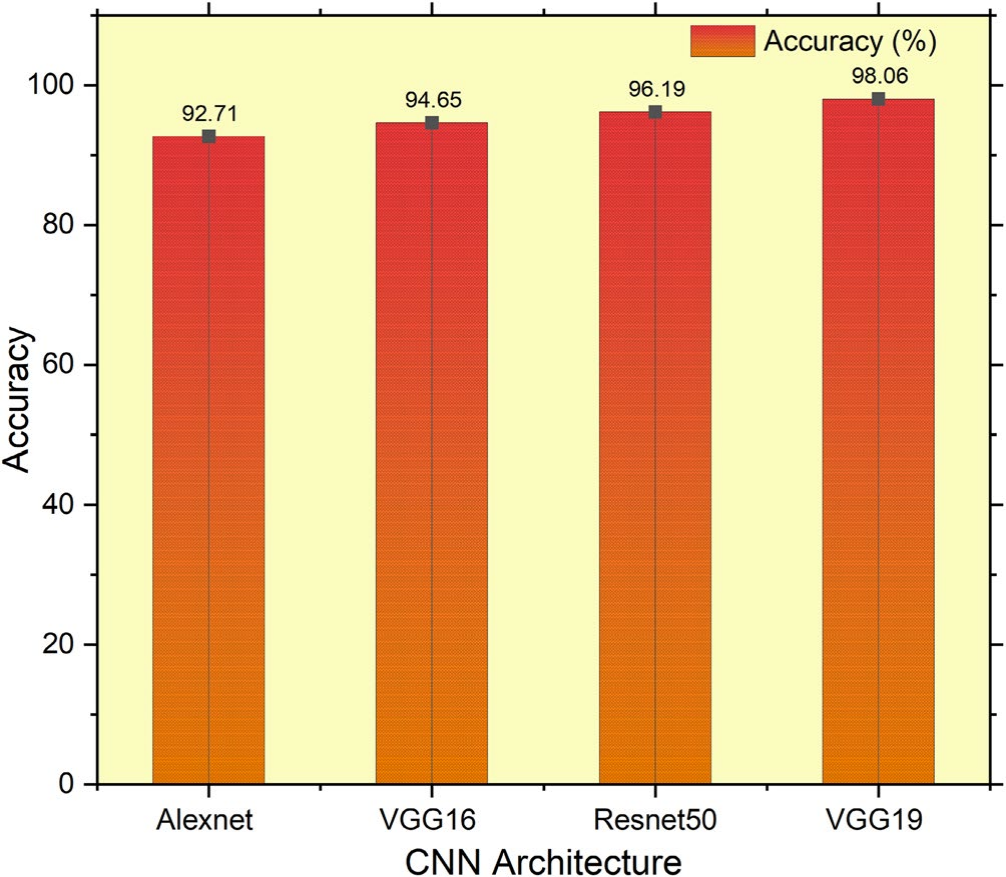
Comparative accuracy analysis using different CNN-based architecture.

Feature extraction is essential to perform accurate Classification of COVID-19 positive and negative. This preliminary analysis helps find the most excellent CNN model for feature extraction. Working on the training and test dataset base as a pre-trained model, VGG19 performs better than other CNN models. Table 2 shows the comparative study of different CNN pre-trained models on the experiment data. All CNN models use a uniform benchmark dataset in training and examine those models over the preprocessed test data set. The classifier head is changed, and the desired model layers are trained to make the difference in the fine-tuned model. However, in this work, the performance of a few scratch models is also computed, but the execution is good enough, like fine-tuned models. For measuring the performance of different CNN models, preprocessed test images are used to extract the test feature. Figure 8 illustrates the accuracy values of different CNN Models, and VGG-19 outperform others in terms of achieving maximum accuracy.

**Table 2:** Comparative results of different CNN models in terms of accuracy, sensitivity, specificity and precision.

Different CNN Models	Accuracy (%)	Sensitivity (%)	Specificity (%)	Precision (%)
AlexNet	92.71	94.11	87.54	93.47
VGG16	94.65	94.69	95.33	93.54
Resnet50	96.19	96.60	93.70	94.09
VGGFusionNet	98.06	97.22	98.97	97.01

**Figure 9:**
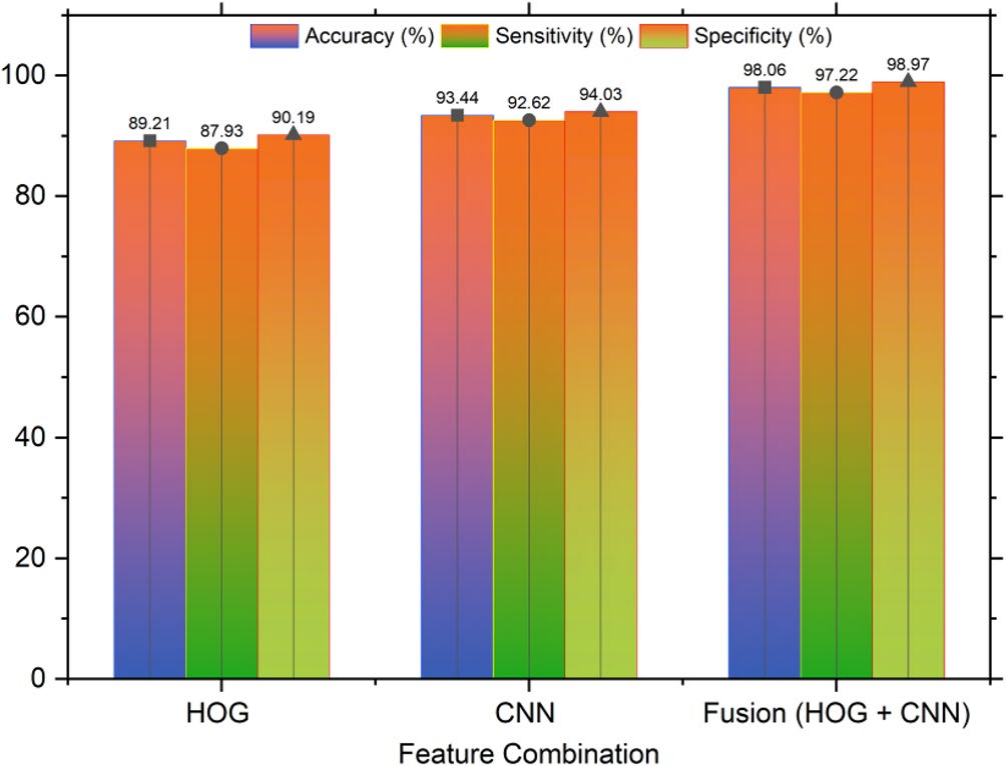
Performance comparison of a fused feature vector.

After deep analysis, the pre-trained model fine-tuned VGG19 is selected for the feature extractor using CNN in this research work. While analyzing the performance of fine-tuned VGG19 and fine-tuned ResNet50, fine-tuned VGG-19 shows a better accuracy of the proposed dataset. However, ResNet50 shows higher sensitivity. The features are extracted for the preprocessing and training purpose from the benchmark dataset in HOG texture. After fusing these textural features and CNN features, a fusion vector is further generated. This fusion is properly considered when creating the training and test vectors. For training the CNN, this training vector is deployed. In Fig. 9, the results of applying various feature fusion combinations are evaluated. In the final classification result, the proposed feature shows the optimal performance.

**Figure 10:**
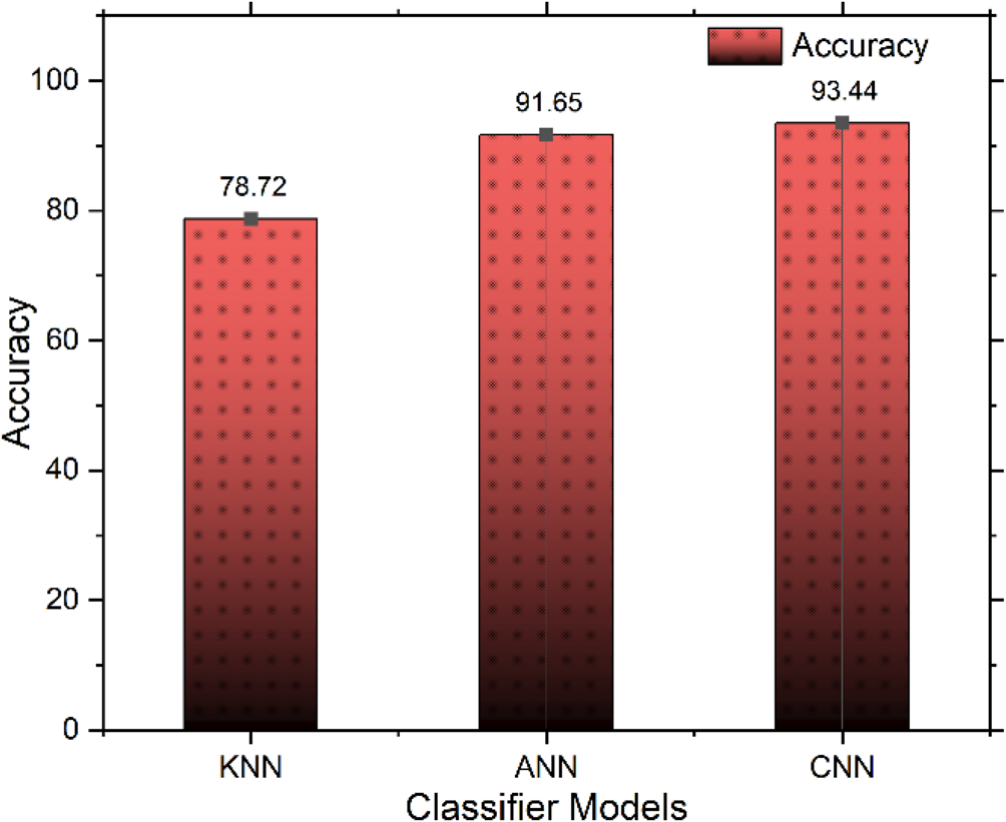
Different classifier models and their accuracy comparison.

Besides the CNN model, two Machine Learning based classifiers are also employed to find the best-suited one. Among the K-nearest neighbour (KNN), Artificial Neural Network (ANN), and Deep learning-based CNN model, CNN architecture shows a better accuracy with almost 93.50%. By using the proposed feature vector, all the classifiers have been evaluated. Classification using different classifier models is tabulated in Table 3, and a graphical illustration is depicted in Fig. 10.

**Table 3:** Obtained results and compared different classifier models in various measures.

Classifier models	Sensitivity	Specificity	False positive rate (FPR)	False negative rate (FNR)
KNN	75.98	80.07	0.24	0.19
ANN	91.79	91.52	0.082	0.085
CNN	92.62	94.03	0.061	0.083

Additionally, to test the robustness of the proposed approach in the detection of COVID-19 patterns, this experiment combined two datasets to train the proposed model and considered another one to test. Thus, the proposed model has investigated the robustness of the performance metrics by a cross-dataset scenario and tabulated the findings in Table 4. From the first dataset (A) 640 samples out of 1700 (37.65% of the total sample) are taken for testing purposes and samples from datasets (B) and (C) are used for training the model. Finally, 87.45% accuracy is obtained. For the second test, the samples of the (A) and (C) datasets are used for training, 49.2% of samples from the dataset (B) are used for testing the model and 91.25% accuracy is obtained. For the last experiment, 12.6% of samples from the dataset (C) are used in testing the model, and samples of (A) and (B) datasets are used for training the model. Finally, 86.97% accuracy is achieved.

**Table 4:** Comparative results of cross dataset scenario.

Test dataset	Accuracy (%)	Sensitivity (%)	Specificity (%)	Precision (%)
Dataset A (640 images)^27^	87.45	84.19	85.63	84.27
Dataset B (400 images)^28^	91.25	89.91	90.58	87.14
Dataset C (312 images)^29^	86.97	83.70	81.59	84.16

## Discussions

COVID-19 is a highly contagious disease. For lessening the transmission rate among people, it is imperative to identify the infected person with COVID-19 to ensure his treatment and proper isolation to curtail the spread of the infection. RT-PCR test on nasopharyngeal scrubs is the fastest and most popular diagnosis method. However, this system does not always serve immediate results, especially considering remote geographical areas and non-developed countries with trivial medical facilities. On the other hand, Computerized Tomography (CT) is becoming a more popular and definitive diagnosis process for COVID-19.

Studies^36,37^ illustrated few circumstantial signs from CT images, and with the help of deep learning image analysis, medical professionals can diagnose a COVID-19 patient more precisely. Our study has accumulated 4861 CT images, including COVID-19 positive and negative, from three different sources to form a dataset randomly divided into a 0.8:0.2 ratio for training and testing our model. We have introduced MADF to remove noise from resized grayscale CT images since it can preserve edges much better than other techniques. At this stage, we have examined several feature extraction methods. A fusion of HOG and VGG19 feature extractors outperformed others. Therefore, our proposed fused feature vector is used in the CNN classifier to get either positive or negative results. Different researchers used different datasets, methods, and machine facilities, so it is not easy to accurately compare. However, some same characteristics are found after observing several existing works then a table is formed to highlight and compare their result with our proposed model. Table 5 shows the comparison result of existing methods to detect COVID-19 with CT images.

**Table 5:** An overview of relevant studies employing deep learning algorithms for COVID-19 case identification, as well as their working procedures and performance metrics.

References	Number of samples	Modality	Method	Validation	Performance metrics
Fang et al.^7^	46 COVID-19, 29 others pneumonia	COVID-19 diagnosis	Radiomic feature, consensus clustering	Random patient-level	AUC = 0.826
Jin et al.^21^	723 COVID-19, 413 others	COVID-19 diagnosis	UNet++, ResNet50	Random patient-level	SEN = 0.97 SPE = 0.92
Xu et al.^38^	110 COVID-19, 224 viral pneumonia, 175 others	COVID-19 diagnosis	3D CNN, 3DResNet	Random patient-level	ACC = 0.86
Song et al.^24^	88 COVID-19, 100 bacterial pneumonia	COVID-19 diagnosis	OpenCV, DRE-Net	Random patient-level	AUC = 0.95
Wang et al.^30^	313 COIVD-19, 229 others	COVID-19 diagnosis	UNet, 3DResN vel	Random patient-level	AUC = 0.98
Li et al.^31^	468 COVID-19, 1551 CAP, 1303 others	COVID-19 diagnosis	UNet, ResNet50	Random patient-level	AUC = 0.96
Wang et al.^39^	4106 lung cancer, 924 COVID-19, 342 others pneumonia	COVID-19 diagnosis, prognosis	FPN, DenseNet	External patient-level	AUC = 0.87, and 0.88
Shibly et al.^40^	1100 normal, 3000 non COVID pneumonia, and 80 COVID-19	COVID-19 diagnosis	2D CNN	Random patient-level	ACC:0.97 SEN:0.97 PREC:0.99
Shi et al.^41^	151 non-severe, 45 severe	Severity diagnosis of COVID-19	V-Net, LASSO, logistic regression	Random patient-level	AUC = 0.89
Shi et al.^42^	1658 COVID-19, 1027 CAP	COVID-19 diagnosis	VBNet, hand-crafted feature, random forest	Random patient-level	ACC = 0.88
Our proposed architecture (VGGFusionNet)	3068 COVID-19 positive 1793 COVID-19 negative	COVID-19 diagnosis	MADF, VGG19, fused HOG and CNN features	Random patient-level	ACC = 0.9806 SPEC = 0.9897 SEN = 0.9722 PREC = 0.9701

Our proposed VGG19 model with fused HOG and deep learning features has acquired 98.06% accuracy and 98.97% specificity with sensitivity and precision of 97.22% and 97.01%, respectively, on our testing data. Analyzing other machine learning and deep learning-based models shown in Table 5, we can state that our proposed model has achieved higher accuracy and can be utilized for Computer-Aided Diagnosis of COVID-19 by medical professionals. Nonetheless, a larger dataset consisting of more random data samples from different regions worldwide for training and testing; may enhance the accuracy of our proposed model. Shortly, we plan to develop a complete web-based real-time diagnosis tool for COVID-19 using our proposed architecture.

## Conclusion

Indisputably, early detection of COVID-19 positive cases is the key to ensuring proper treatment and, most importantly, inhibiting current outbreaks worldwide. Our proposed feature fusion-based deep learning model is an intelligent system to detect COVID-19 precisely from chest CT images and a progression to accelerate the COVID-19 test count significantly by asserting the proficiency of differentiating COVID-19 positive and negative of CT images with the help of a deep learning model. Moreover, frontline medical professionals can be aided by this system to diagnose COVID-19 even with the slightest manifestation in the human lungs.

## Data Availability

The data supporting this study's findings are available from the corresponding author upon reasonable request.
